# Where to Meet

**Published:** 1886-04

**Authors:** 


### EDITORIAL.

### Where to Meet.

The settlement of the question, where shall the next meeting of the American Dental Association be held ? is enlisting a good deal of interest just now, and some active work as well. At the last meeting, the selection of the place of the next meeting was left in the hands of the Executive Committee. The Committee have become divided in the choice of place. Within a few weeks, the way has opened for cheap travel to California. Of course almost every body wishes and expects to go to California some time.

Dentists doubtless as much as any other class of people, share this desire, and to them, the opportunity seems more inviting now, than ever before. There are now unprecedentidly low railroad fares. A very cordial and urgent invitation is extended for the meeting by the profession of California, and to meet with them would be very pleasant indeed, and give a rare opportunity for greeting friends of former days.

For several years anticipations have been entertained by many that the time would soon come when it would be practicable for the Association to meet on the Pacific slope, the present is the most favorable time that has as yet occurred, and perhaps as favorable as will occur in the near future.

There is, however, some objection as to the time of the year. It would be better to go in May or November, rather than in midsummer; and after all there is difference of opinion on this point. The journey in the summer may be dusty and warm, or it may not, as we have been informed by those who have been often over the road. The summer season in California, is for the most part very pleasant; and the travel through the mountainous parts much more practicable and pleasant in the summer than in the winter or wet season.

The probability is that if the Association is not held in California, the profession of the East will not be satisfied to have it held this year farther West than Detroit or, perhaps better, Niagara Falls. It was in the far West last year, and should not be taken there again, except to an entirely new point, and by the general consent of those interested. It would seem that the most natural and reasonable arrangement for the meetings of the Association, would be to meet once every three years at it birth place—Niagara Falls, and the other years, alternate between the East and the West, going more or less North or South, as the best interest might seem to demand. In no instance should the Association be held more than once, or at most twice, in the same place, except Niagara Falls, without some special reason that would commend itself to the judgment and approval of a large majority of the members.

Were it not that it will not be practicable for the Association to meet in San Francisco in 1887, the meeting ought to be held at Niagara this year.

It probably did not occur to those who organized this Association and framed its constitution, that the variety of climates of this immense country, would modify the time of meeting if a reasonable degree of comfort and pleasure are to be enjoyed by those in attendance.

